# Mean Cholinesterase Level among Organophosphorus Poisoning Patients Visiting the Emergency Department in a Tertiary Care Centre: A Descriptive Cross-sectional Study

**DOI:** 10.31729/jnma.7983

**Published:** 2023-01-31

**Authors:** Binita Pradhan, Sujan Pandey, Aliska Niroula, Nishob Adhikari, Nibedita Chapagain, Sailesh Pradhan

**Affiliations:** 1 Department of Emeraency, Kathmandu Medical Colleae and Teachina Hospital, Sinamanaal, Kathmandu, Nepal; 2 Kathmandu Medical Colleae and Teachina Hospital, Sinamanaal, Kathmandu, Nepal; 3 Department of Patholoay, Kathmandu MedicalColleae and Teachina Hospital, Sinamanaal, Kathmandu, Nepal

**Keywords:** *cholinesterases*, *liver function tests*, *organophosphorus poisoning*

## Abstract

**Introduction:**

Acute organophosphorus pesticide poisoning is widespread and the most common in many developing countries, including Nepal. Through the inhibition of acetylcholinesterase, organophosphorus poisoning is characterised by the clinical picture of acute cholinergic crisis. Many researchers have shown increased levels of liver enzymes and decreased levels of serum cholinesterase in organophosphorus poisoning, however, very little work has been done in Nepal that studies the correlation between serum cholinesterase and liver enzymes in organophosphorus poisoning. The aim of the study is to find out the mean cholinesterase level among organophosphorus poisoning patients visiting the Emergency Department in a tertiary care centre.

**Methods:**

This was a descriptive cross-sectional study done among 94 organophosphate poisoning cases visiting the Emergency Department of a tertiary care centre from August 2021 to August 2022 after obtaining approval from the Institutional Review Committee (Reference number: 04102021/06). Convenience sampling was done. Blood work up were done for cholinesterase and liver function tests. Point estimate and 90% Confidence Interval were calculated.

**Results:**

The mean cholinesterase level among organophosphorus poisoning patients was 1.97±1.87 U/ml (1.66-2.29, 90% Confidence Interval).

**Conclusions:**

The mean cholinesterase level among organophosphorus poisoning patients was similar when compared to other studies done in similar settings.

## INTRODUCTION

Organophosphates (OP) are used as insecticides in agricultural and domestic settings.^1^ Along with causing environmental pollution, the organophosphate compound is also the major cause of acute and chronic OP poisoning.^2^ It is a common problem, particularly in developing countries. Around 3 million cases of pesticide poisoning occur every year, of these about 1 million are accidental and 2 million are of suicidal intentions.^3^

OP compounds inhibit acetylcholinesterase (AChE) and plasma cholinesterase (PChE) enzymes. The irreversible inhibition of these enzymes leads to the accumulation of acetylcholine and subsequent over activation of cholinergic receptors in various parts of the body.^4^ The organophosphorus compounds generate free radicals which may alter the liver metabolism and are evidenced by changes in the level of its enzymes.^5^

The aim of the study was to find out the mean cholinesterase level among organophosphorus poisoning patients visiting the Emergency Department in a tertiary care centre.

## METHODS

A descriptive cross-sectional study was conducted among all the organophosphorus poisoning cases brought to the Department of Emergency, Kathmandu Medical College and Teaching Hospital (KMCTH) from August 2021 to August 2022 after obtaining approval from the Institutional Review Committee of KMCTH (Reference number: 04102021/06). Convenience sampling was done. The inclusion criteria were patients visiting the Emergency Department of KMCTH. The study's exclusion criteria were the patients not giving consent to the study. The sample size was calculated using the following formula:


$$\begin{array}{ll} \text{n} & ={\text{Z}}^{2}\times \frac{\sigma^{2}}{{\text{e}}^{\text{2}}}\\ & =1.64^{2}\times \frac{0.8317}{0.0141^{2}}\\ & =94 \end{array}$$

Where,

n = minimum required sample size
Z = 1.64 at 90% Confidence Interval (CI)
σ = Standard Deviation (SD) of cholinesterase level^6^
e = margin of error

A questionnaire was formed to collect the patients' data, including the hospital number, age, sex, vitals, cholinesterase level, and liver function test. Serum cholinesterase levels were analyzed by using the crosslinked enzyme aggregate method and the liver function test analysed by biochemistry analyzer was collected. Data were analyzed using IBM SPSS Statistics version 21.0. Point estimate and 90% CI were calculated.

## RESULTS

**Figure 1:**
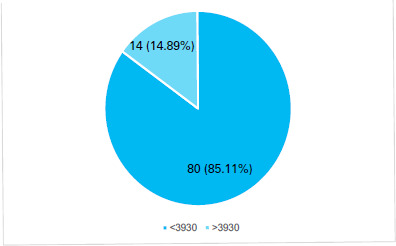
Serum cholinesterase level (n= 94).

The mean cholinesterase level among organophosphorus poisoning patients was 1.97±1.87 U/l (1.66-2.29, 90% Confidence Interval). Serum cholinesterase level was found to be low (<3.93 U/ml) in 80 ( 85.11%) (Figure 1).

The descriptive statistics of age, vitals and laboratory parameters of the patients with OP poisoning (Table 1).

**Table 1:** Descriptive statistics of patients with OP poisoning (n= 94).

Parameters	Mean±SD
Age (years)	32.05±15.17
Pulse	89.18±16.30
Systolic BP	118.51±14.06
Diastolic BP	74.57±9.57
RR	21.03±3.31
Temperature	98.49±1.61
SaO_2_	90.19±5.625
GCS	13.70±1.825
Cholinesterase	1978.82±1878.22
TSB (mg/dl)	0.828±0.28
DB (mg/dl)	0.398±0.172
SGPT (U/L)	38.14±25.333
SGOT (U/L)	38.50±23.806
ALP (U/L)	281.67±181.223

Out of 80 low cholinesterase levels, 65 (81.25%) patients had raised serum glutamic pyruvic transaminase (SGPT) and 25 (31.25%) had raised serum glutamic-oxaloacetic transaminase (SGOT) (Table 2).

**Table 2:** Liver enzymes among patients with low cholinsterase level (n= 80).

Liver enzymes	n (%)
Raised total serum bilirubin	5 (6.25)
Raised direct bilirubin	24 (30)
Raised SGPT	65 (81.25)
Raised SGOT	25 (31.25)
Raised ALP	80 (100)

Age group of 20-30 years was found to be the commonest age group for organophosphorus poisoning comprising 28 (29.79%) followed by 30-40 years age group 22 (23.40%). Total of 48 (51.06%) were females with female:male ratio of 1:0.96 (Table 3).

**Table 3:** Socio-demographic details of the OP poisoning cases (n= 94).

Variables		n (%)
	0-10	4 (4.26)
	10-20	17 (18.09)
	20-30	28 (29.79)
Age group (years)	30-40	22 (23.40)
	40-50	11 (11.70)
	50-60	9 (9.57)
	60-70	2 (2.13)
	70-80	1 (1.06)
Sex	Female	48 (51.06)
	Male	46 (48.94)

**Figure 2:**
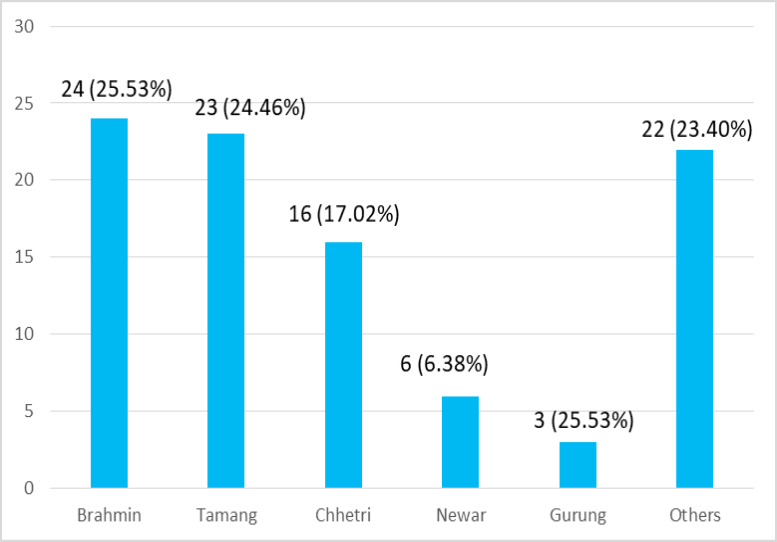
Ethnicity distribution of cases of OP poisoning (n= 94).

Among 94 cases, there were 24 (25.53%) Brahmin patients and 23 (24.47%) Tamang patients (Figure 2).

## DISCUSSION

OP poisoning is a major issue in the context of developing countries like Nepal, with increasing prevalence, morbidity and mortality across developing countries like Nepal. The mean cholinesterase level among organophosphorus poisoning patients was 1.97±1.87 U/ml which was similar to a similar study done in Nepal.^2^

Female predominance was found in our study which is similar to other studies.^2,7-13^ The majority (29.79%) of OP poisoning cases were of the age group 20-30 years in our study which is similar to other studies where OP poisoning was found to be more common among the younger generation.^2^ According to a study done in Nepal, plasma cholinesterase level was found to be significantly decreased in patients with organophosphate poisoning which was similar to our study.^2^

SGOT was found to be elevated in only 26.60% of patient with low cholinesterase however SGPT was found to be raised in 69.15% and 100% case with low cholinesterase have raised ALP.

This study showed a decrease in serum Cholinesterase levels, in patients who had raised SGPT, SGOT and ALT which was similar to other studies.^2^ Similarly, other studies have also shown abnormal liver function tests like a decrease in serum cholinesterase levels and increased AST and ALP levels.^14^ There is elevated liver enzymes in moderate and severe cases of OP poisoning in some studies.^15,16^

This study had some limitations like-small sample size and the study involving a single centre could not generalize the entire population of the nation. Also, the association between the various variables such as gender, age, the quantity of the poison ingested by patients, arrival time since exposure, treatment received and the outcome could not be made in this study. Risk factors could not be made out as well. Further higher studies with a larger number of patients are recommended.

## CONCLUSIONS

The mean cholinesterase level among organophosphorus poisoning patients was similar when compared to other studies done in similar settings. Frequent reports of the poisoning among younger age groups and females could reflect their vulnerability. Since inhibition of cholinesterase is the major mechanism of OP poisoning, level of cholinesterase is important for early diagnosis of organophosphate exposure or intoxication.
